# The *C. elegans* mapping locus *rol-9* is encoded by a gain-of-function mutation in *mlt-11*

**DOI:** 10.17912/micropub.biology.000506

**Published:** 2022-01-05

**Authors:** Matthew S Rich, Paola Nix, Erik M Jorgensen

**Affiliations:** 1 School of Biological Sciences, Howard Hughes Medical Institute, University of Utah, USA

## Abstract

We mapped *rol-9* to the *mlt-11* locus (encoded by the gene W01F3.3) on the far-right end of chromosome V. The canonical allele of *rol-9*, *sc148*, is an in-frame deletion in a conserved exon of the protein that creates a gain-of-function roller phenotype. *sc148* deletes a short peptide of unknown function conserved in nematodes.

**Figure 1. rol-9(sc148) is a gain of function allele of mlt-11:**
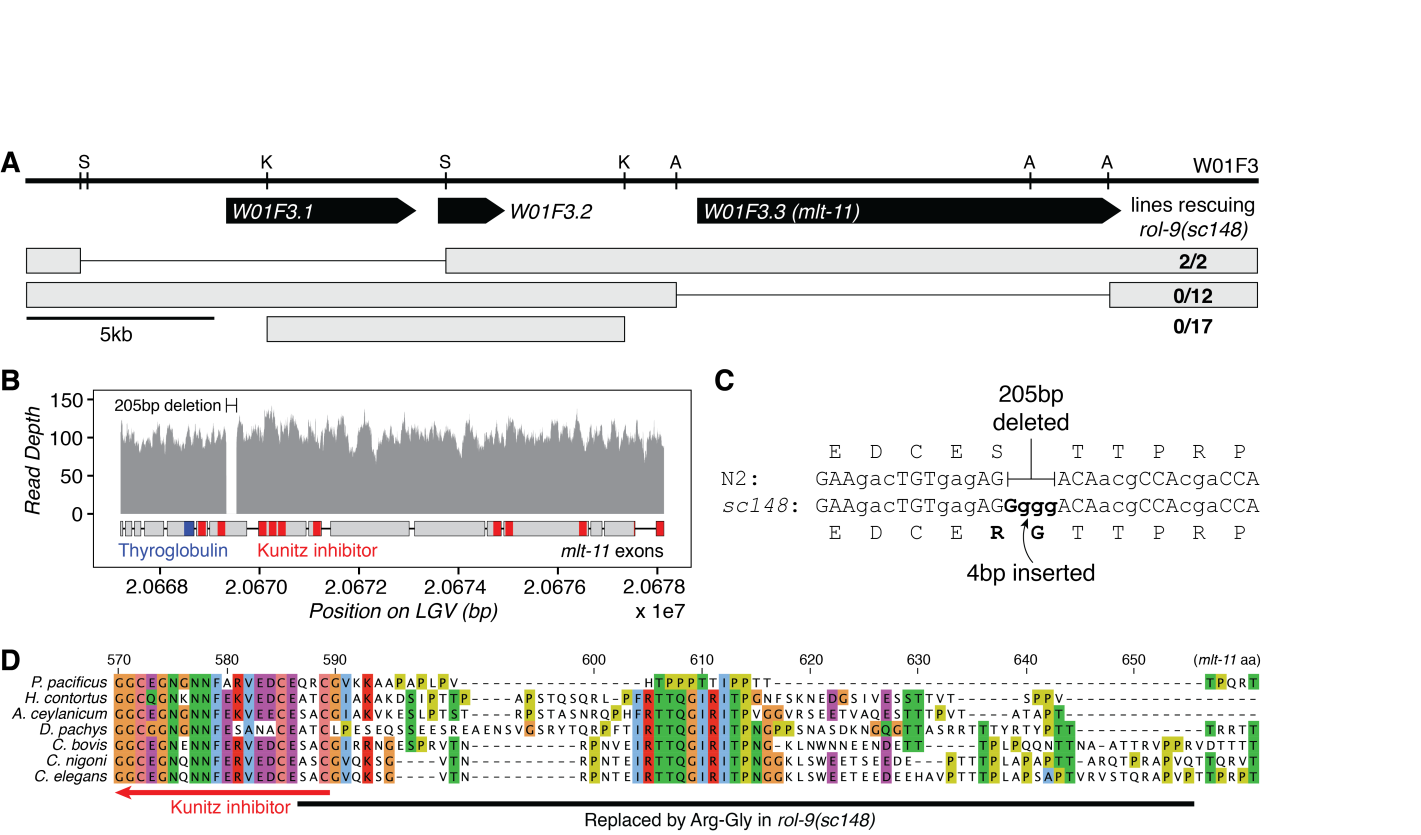
(A) Fosmid rescue experiments define W01F3.3 as the gene encoding *rol-9.* The structure of fosmid W01F3 is shown, with restriction enzyme sites (S: SacI, K: KpnI, A: AatII) and protein-coding genes annotated. Below, representations of restriction fragments are shown with their rates of rescue shown at the right side. Full bars represent the sequence retained in the fragment. (B) *sc148* is a homozygous deletion in *mlt-11.* The deduplicated read depth across the *mlt-11* locus is shown as grey bars. A 205 basepair deletion in *mlt-11* is noted. Below, a representation of the gene structure in *mlt-11* is annotated with the positions of functional domains. (C) *sc148* replaces 205bp with GGGG, making an in-frame replacement of 68aa with Arg-Gly. The wildtype and mutant sequences flanking *sc148* are shown. (D) *sc148* deletes a peptide conserved in Rhabditina nematodes. Shown is a multiple sequence alignment of *mlt-11* orthologs in 7 species of nematodes. The positions of an upstream Kunitz inhibitor domain and the sequence deleted in *sc148* is annotated below the alignment. Amino acid positions are numbered based on *C. elegans mlt-11*.

## Description

*rol-9* has long been used as a marker for the far-right end of chromosome V, but the identity of the protein encoded *rol-9* is unknown. The canonical allele of the gene, *sc148*, is a strong semi-dominant right-hand roller (Bergmann *et al.*, 1998). Another uncharacterized transposon insertion allele of *rol-9*, *zu156* is also a strong roller (Susan Mango and Jim Priess, personal communications).

We mapped and cloned *rol-9* while cloning *pbo-5*, the last gene on LGV (Beg *et al.*, 2008). Injection of the fosmid W01F3 rescued *rol-9(sc148)* (EG1920).We then fragmented W01F3 by restriction digest and identified the gene encoding *rol-9* to be W01F3.3 (Figure 1A). W01F3.3 encodes *mlt-11,* a 340kDa predicted serine endopeptidase inhibitor composed of one thyroglobulin repeat and ten Kunitz inhibitor domains (Frand *et al.*, 2005). Knockdown of *mlt-11* by RNAi induces semi-penetrant embryonic or L1 arrest (Gottschalk *et al.*, 2005; Simmer *et al.*, 2003; Sönnichsen *et al.*, 2005) as well as defects during larval molting, for which the gene was named. Since *rol-9(sc148)* causes a semi-dominant roller, it is likely that the allele is a gain-of-function mutation, and not a null or hypomorphic allele of *mlt-11.*

We precisely mapped the mutation causing *rol-9(sc148)* using whole genome sequencing. There was only one mutation annotated to have high or moderate functional effect on the far-right arm of LGV: a 205 basepair deletion (V:20,669,331-20,669,536, Figure 1B) combined with a four-base insertion (GGGG, Figure 1C). The mutation is found in exon 7 of *mlt-11,* which is conserved in all splice isoforms*,* and replaces 68 residues with an arginine and glycine.

The deletion overlaps only 3 amino acids of a Kunitz-type inhibitor domain in the protein, and the deleted sequence matches no conserved domains in the NCBI Conserved Domain Database (Marchler-Bauer *et al.*, 2015). A 13aa peptide in the deletion is highly conserved in the Rhabditina clade (Figure 1D). This peptide (IRTTQGIRITPNG) is almost perfectly conserved in *Caenorhabditis,* *Haemonchus,* and *Ancylostoma,* but is not conserved in *Pristionchus*. In all the species studied, this peptide is the only conserved sequence between the second and third Kunitz protease inhibitor domains. It is likely that the peptide was introduced into *mlt-11* in the most recent common ancestor of *Haemonchus* and *Caenorhabditis*, after the split with *Pristionchus.*

Most genes with a roller phenotype encode structural constituents of the cuticle or collagens. It isn’t clear how *sc148*, a gain of function mutation in MLT-11, a molting protease inhibitor, induces a Rol phenotype, though it seems likely that the conserved peptide deleted in *sc148* may be playing a Rol.

## Methods

*Fosmid rescue. rol-9(sc148)* adult hermaphrodites were injected with either the full-length W01F3 fosmid or restriction fragments in which the fosmid was cut with either SacI, KpnI, or AatII. Fosmids were co-injected with a fluorescent marker to identify lines with extrachromosomal array transmission into the F2 generation. Transmitting lines were scored for rescue. EG1920 contains an extrachromosomal array containing the full-length W01F3 fosmid.

*Whole genome sequencing and data analysis. rol-9(sc148)* animals were grown on HB101 plates until near starvation, at which point genomic DNA was extracted using a Qiagen DNeasy Blood and Tissue kit. Genomic DNA libraries were prepared using a Nextera transposase kit (Illumina) and were sequenced on an Illumina NovaSeq with 2×150 base reads. Library prep and sequencing were performed by the Huntsman Cancer Institute Genomics Core facility. Alignment and analysis of sequencing reads was performed by an in-house software pipeline. Briefly, reads were aligned to the *C. elegans* genome (WBcel276) using bwa (Li and Durbin, 2009) and processed with samtools (Li, 2011). Basecalling was performed using GATK4 (McKenna *et al.*, 2010), and homozygous mutations with high genotype quality (GQ>20) were annotated with SNPeff (Cingolani *et al.*, 2012). We identified the deletion in W01F3.3 algorithmically using smoove (Pedersen *et al.*, 2020) and by eye using the Integrative Genome Viewer (Robinson *et al.*, 2011).

*Conservation analysis.* Nematode orthologs of *mlt-11* were identified using BLASTP (Altschul *et al.*, 1990). Potential hits were defined as having an E-value less than 1e-100 and a protein length greater than 3000aa. The full-length sequences of these proteins were aligned using Clustal Omega (Goujon *et al.*, 2010; Sievers *et al.*, 2011). The sequences used in the alignment are: *Caenorhabditis elegans* (NP_001256938.1)*, Caenorhabditis nigoni* (PCI30935.1), *Caenorhabditis bovis* (CAB3399020.1), *Diploscapter pachys* (PAV69501.1), *Ancylostoma ceylanicum* (EPB71358.1), *Haemonchus contortus* (CDJ84543.1), and *Pristionchus pacificus* (KAF8386888.1).

## Reagents

The *rol-9(sc148)* allele in EG10047 was isolated as a segregant of a mapping cross (Beg *et al.*, 2008). EG1920 contains an extrachromosomal array that rescues both *rol-9(sc148)* and *pbo-5(ox9).*

**Table:** 

Strain	Genotype	Source
EG10047	*rol-9(sc148) V*	this study
EG1920	*rol-9(sc148) pbo-5(ox9) V*; *oxEx266*[*Psur-5*::GFP, W01F3, purified yeast genomic DNA containing Δ200 (Y44A6 deletion from 5′ end of Y44A6A to 3′ end of W07A8)]	Beg *et al.* 2008
